# Development of Variable Charge Cationic Hydrogel Particles with Potential Application in the Removal of Amoxicillin and Sulfamethoxazole from Water

**DOI:** 10.3390/gels10120760

**Published:** 2024-11-23

**Authors:** Francisca L. Aranda, Manuel F. Meléndrez, Mónica A. Pérez, Bernabé L. Rivas, Eduardo D. Pereira, Daniel A. Palacio

**Affiliations:** 1 Departamento de Ingeniería de Materiales, Facultad de Ingeniería, Universidad de Concepción, Concepción 4070371, Chile; 2 Departamento de Polímeros, Facultad de Ciencias Químicas, Universidad de Concepción, Concepción 3349001, Chile; 3 Facultad de Ciencias para el Cuidado de la Salud, Universidad San Sebastián, Campus Las Tres Pascualas, Concepción 4060000, Chile; 4 Departamento de Química Analítica e Inorgánica, Facultad de Ciencias Químicas, Universidad de Concepción, Casilla 160-C, Concepción 3349001, Chile

**Keywords:** cationic hydrogels particles, wastewater treatment, adsorption mechanism, antibiotics, pharmaceutical compounds

## Abstract

Cationic hydrogel particles (CHPs) crosslinked with glutaraldehyde were synthesized and characterized to evaluate their removal capacity for two globally consumed antibiotics: amoxicillin and sulfamethoxazole. The obtained material was characterized by FTIR, SEM, and TGA, confirming effective crosslinking. The optimal working pH was determined to be 6.0 for amoxicillin and 4.0 for sulfamethoxazole. Under these conditions, the CHPs achieved over 90.0% removal of amoxicillin after 360 min at room temperature, while sulfamethoxazole removal reached approximately 60.0% after 300 min. Thermodynamic analysis indicated that adsorption occurs through a physisorption process and is endothermic. The ΔH° values of 28.38 kJ mol^−1^, 12.39 kJ mol^−1^, and ΔS° 97.19 J mol^−1^ K^−1^, and 33.94 J mol^−1^ K^−1^ for AMX and SMX, respectively. These results highlight the potential of CHPs as promising materials for the removal of such contaminants from aqueous media.

## 1. Introduction

Water is an essential and indispensable resource for any ecosystem [1,2]. However, it is currently severely impacted by domestic wastewater, agricultural and industrial residues, and pharmaceutical compounds [3,4]. Among these, emerging organic contaminants such as antibiotics have become a major concern. It is estimated that between 20% and 90% of the total antibiotics consumed are not metabolized and are excreted either unchanged or as derivatives and metabolites, which directly enter aquatic systems [5,6].

**Figure 1:**
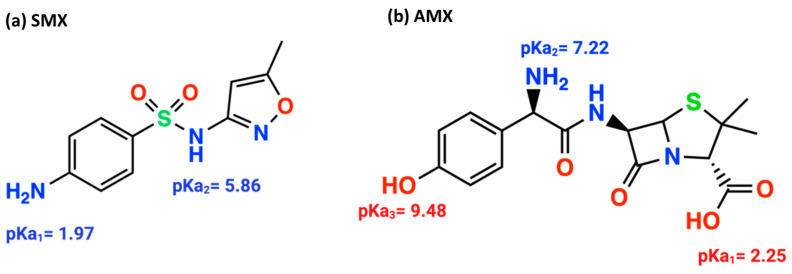
Structures of the antibiotics sulfamethoxazole (**a**), amoxicillin (**b**), and their respective pKa values.

The most widely used antibiotic families worldwide include β-lactams and sulfonamides, particularly amoxicillin and sulfamethoxazole, respectively. Amoxicillin, a penicillin derivative, has 6-aminopenicillanic acid as its core structure, consisting of a thiazolidine ring fused with a β-lactam ring and a side chain. This structure contains three main functional groups (Figure 1b): COOH (pKa_1_ = 2.25), NH_2_ (pKa_2_ = 7.22), and OH (pKa_3_ = 9.48) [7,8,9]. Sulfamethoxazole, used to treat various diseases and infections [10], contains a basic amine group (-NH_2_) and an acidic sulfonamide group (-SO_2_NH-), resulting in two distinct dissociation sites within the molecule (Figure 1a), with pKa_1_ and pKa_2_ values of 1.97 and 5.86, respectively [11,12].

Currently, a wide variety of materials are available for the removal of such contaminants [7,13], including carbon-based materials and metal–organic frameworks (MOFs) with removal efficiencies ranging from 75.3% to 82.52% [14,15]. Other compounds, such as In_2_S_3_, achieve 66% removal [16], while materials like UiO-66@WO_3_/GO exhibit an 84% removal rate [17]. Porous organic polymers, such as triazine-based materials, demonstrate removal efficiencies between 73% and 88% [18].

Among polymeric materials with promising applications, chitosan [(1,4)-2-amino-2-deoxy-D-glucan] stands out. Chitosan is a polysaccharide derived from chitin, one of the most abundant natural substances after cellulose, and serves as the primary structural component of the exoskeletons of shrimp, lobsters, and crabs [19]. It exhibits excellent biological properties and finds extensive applications in medical and food sectors. Chemically, chitosan is a cationic polysaccharide composed of glucosamine units linked by glycosidic bonds. Its biocompatibility, biodegradability, and antibacterial activity further enhance its versatility [20,21,22]. Chemical modification of chitosan improves its absorption properties, solubility, porosity, and permeability. Its high nitrogen content, present as amine groups, enables interactions with various compounds through chelation mechanisms, offering bifunctional capabilities for the removal of both cationic and anionic contaminants [23,24].

Chitosan can undergo various chemical modifications, including etherification, carboxylation, crosslinking, alkylation, and the formation of Schiff bases, enhancing its antibacterial properties, hydrophilicity, and water solubility [25]. Due to its versatility, chitosan is widely used in wastewater treatment as an additive or in composites with other materials such as montmorillonite, polyurethanes, zeolites, cellulose, magnetite, cotton, calcium alginate, and alumina, among others.

A variety of chitosan-based materials have been reported for contaminant removal. For instance, chitosan nanocomposites with MnFe_2_O_4_ nanoparticles have shown removal capacities of 20.85 mg g^−1^, while those with Fe_3_O_4_ nanoparticles have achieved 78.11 mg g^−1^ [26]. Photocatalytic removal of sulfamethoxazole has also been reported using chitosan/alginate nanocomposites doped with Fe_3_O_4_/ZnO, with antibiotic degradation rates of 93.31% [27]. The employment of cationic hydrogel particles (CHP) based on chitosan crosslinked with glutaraldehyde for the removal of pharmaceutical antibiotics, specifically amoxicillin and sulfamethoxazole from aqueous solutions is a promising approach. This is due to the greater advantages cationic materials exhibit over other materials. This is a consequence of the tendency of antibiotics to ionize, which increases their interaction with the active sites of the particles. Concurrently, they can interact with polar groups, thereby enhancing the efficacy of contaminant removal through van der Waals interactions. Consequently, CHPs demonstrate considerable potential for the elimination of emerging organic pollutants in water, outperforming other materials.

## 2. Results and Discussion

### 2.1. Synthesis of Cationic Hydrogel Particles (CHPs)

**Figure 2:**
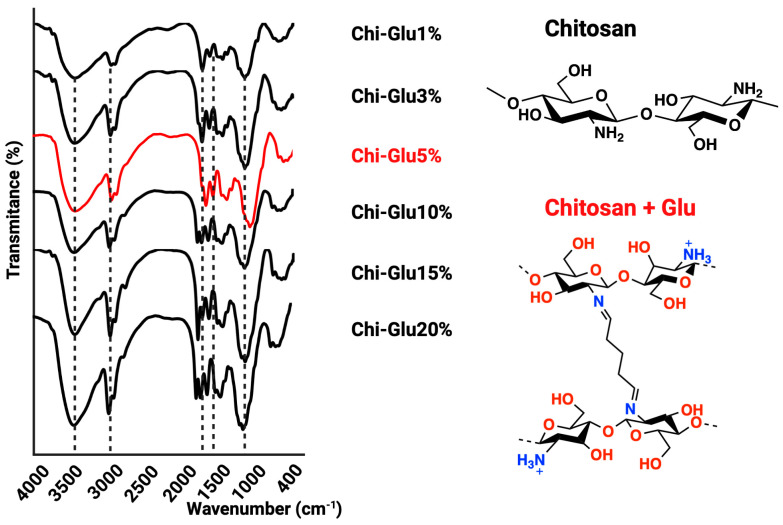
FTIR spectra of CHPs.

The synthesis of CHPs was conducted in accordance with the methodology delineated in Table 2. The NaOH solution was delivered via a peristaltic pump, and once the CHPs were formed, the crosslinking agent, glutaraldehyde (Glu), was introduced under agitation [28]. The crosslinking capacity of Glu is attributed to the nucleophilic reaction between its aldehyde groups and the free amino groups of chitosan, enhancing the mechanical, thermal, and water resistance properties of the resulting hydrogel [29,30,31]. The Glu concentration was set at 5%, as determined by FTIR spectra (Figure 2), which showed the availability of active amino groups and a decrease in the intensity of the band associated with these groups as the Glu concentration increased; furthermore, an elevated Glu concentration enhances the band signal of carbonyl and amine groups associated with an excess of Glu. The FTIR spectra of the CHPs displayed the following characteristic bands: 3437 cm^−1^ (N–H and O–H stretching vibrations), 2925 cm^−1^ (symmetric stretching of CH_3_), 1660–1670 cm^−1^ (C=O stretching vibration), and 1150 cm^−1^ (glycosidic bond) [32].

**Figure 3:**
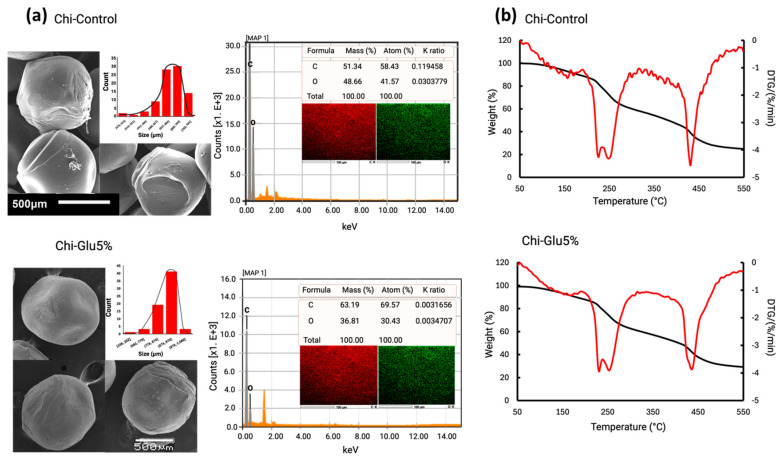
(**a**) SEM-EDS microstructure of CHIPs (Chi-Control and Chi-Glu5%) and size distribution; (**b**) thermogravimetric analysis of CHIPs (Chi-Control and Chi-Glu5%).

Between 1560 and 1600 cm^−1^, an increase in intensity is observed, associated with the C=C double bond, attributed to the aldol condensation of the Glu molecule [33,34,35]. The application of glutaraldehyde-mediated crosslinking resulted in an observable increase in the signals between 1700 and 1740 cm^−1^, which can be attributed to the C=O signal of glutaraldehyde. This phenomenon indicates that as the concentration of glutaraldehyde increases, A greater proportion of these groups remain unreacted, as evidenced by the presence of signals between 1630 and 1690 cm^−1^. These are associated with the imine bonds (C=N) formed by the reaction of the amino groups of chitosan and glutaraldehyde [36]. The morphological characteristics of the CHP are illustrated in Figure 3a. These studies reveal a rough surface with a spherical geometry, which is predominantly populated by particles measuring between 874 and 970 µm for the crosslinked CHPs, while control CHPs (Chi-Control) are predominantly by particles measuring between 600 and 700 µm. The difference can be attributed to the glutaraldehyde employed. The surface roughness of the CHPs provides an increased number of adsorption sites, enhancing the removal of the target compounds [32]. Furthermore, the roughness is closely related to the degree of crosslinking.

Figure 3b shows the thermogravimetric analysis (TGA) of the CHPs (Chi-Control and Chi-Glu5%), revealing that the crosslinked CHPs (Chi-Glu5%) exhibit greater thermal stability than the non-crosslinked ones, with two distinct stages of mass loss. The first stage shows a slight mass loss associated with the evaporation of adsorbed and chemically bound water in the structure of the CHPs. It is also noted that non-crosslinked particles experience greater water loss, while the crosslinked CHPs retain less water due to the hydrophobic nature acquired after the crosslinking reaction. The derivative thermogravimetry (DTG) results indicate that the thermal decomposition temperature of the Glu-crosslinked CHPs occurs at a higher temperature, attributed to the formation of a chemically crosslinked network, which improves the material’s thermal stability [37,38]. In the second stage, decomposition is observed in both the control and crosslinked CHPs. Although greater decomposition is seen in the control material, and the residual mass is lower compared to the Glu-crosslinked material, no significant difference is observed. The residual percentages of the crosslinked and non-crosslinked beads are found to be similar, which can be attributed to the low concentration of glutaraldehyde employed (5%). The quantity in question is insufficient to affect a notable alteration in the material’s structure.

### 2.2. Determination of Water Absorption Capacity

**Figure 4:**
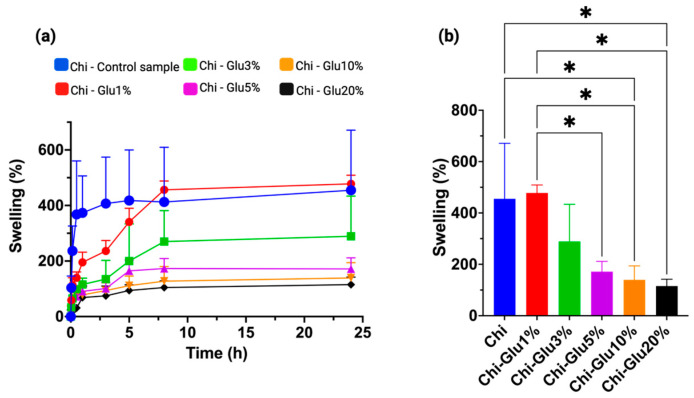
(**a**) CHPs crosslinked with glutaraldehyde at different concentrations subjected to a water absorption process over time and (**b**) comparison of water absorption at equilibrium time 8 h. * Significant at *p* < 0.01.

The absorption and diffusion of water in polymeric materials are influenced by the degree of molecular crosslinking [39]. As shown in Figure 4a, lower concentrations of glutaraldehyde result in higher water absorption. The maximum absorption equilibrium was reached after 8 h, with no significant changes observed beyond this point. This behavior is attributed to the crosslinking process between amino groups and Glu, which reduces the material’s interaction with water as the concentration of the crosslinking agent increases [40,41]. This phenomenon likely occurs due to a reduction in the internal cavities of the material, limiting the diffusion of water into the interior and resulting in decreased water absorption.

### 2.3. Evaluation of pH Effect on Removal and Point of Zero Charge (PZC)

**Figure 5:**
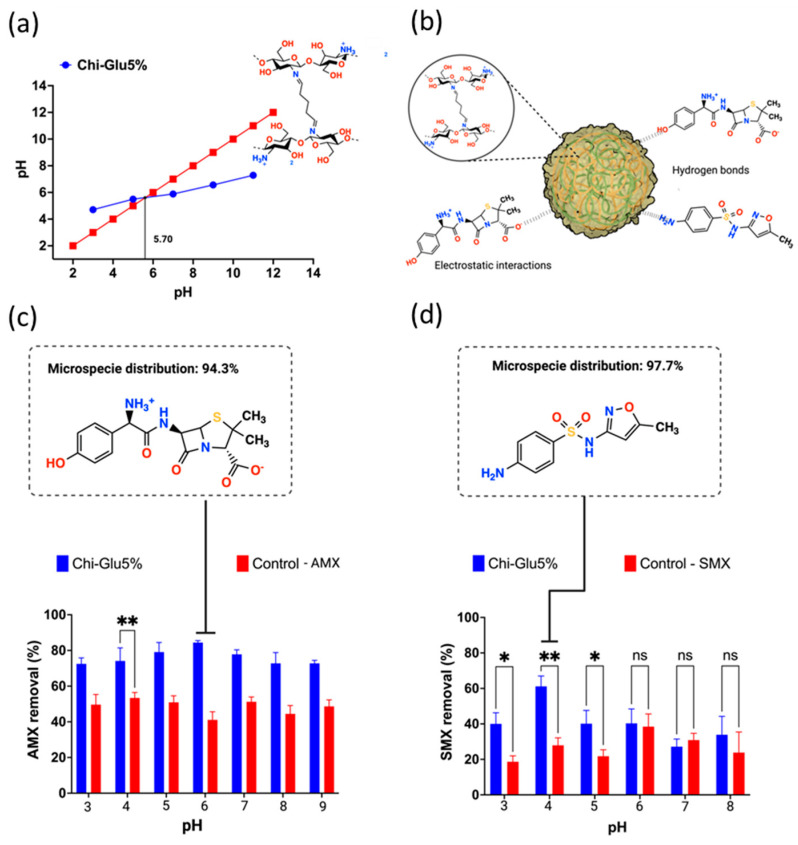
(**a**) Identification of the point of zero charge (PZC) of chitosan CHPs; (**b**) adsorption mediated by electrostatic interactions and hydrogen bonding between the crosslinked chitosan CHPs and the study antibiotics; removal of the antibiotics (**c**) amoxicillin and (**d**) sulfamethoxazole at different pH values. ** Significant at *p* < 0.05, * Significant at *p* < 0.01, ns = no significant difference.

By definition, the point of zero charge (PZC) is the pH at which the surface charge of the adsorbent is neutralized [42]. For CHPs, the PZC was determined to be 5.7 (Figure 5a), meaning that at this pH, the surface charge of the CHPs is zero, and no electrostatic repulsions occur due to the absence of charged particles. At pH values below the PZC, protonation of surface hydroxyl groups and available amino groups occurs, resulting in a positively charged surface. Conversely, at pH values above the PZC, deprotonation of hydroxyl groups takes place, leading to a negatively charged surface [43].

Figure 5c,d shows the effect of pH on the adsorption capacity of crosslinked CHPs for the antibiotics AMX and SMX, respectively, with a contact time of 6 h at room temperature. The highest adsorption of AMX occurs at pH 6.0, while for SMX, it is at pH 4.0. Considering that the PZC is 5.7, at pH values below this point, the surface of the CHPs becomes positively charged. Additionally, it is important to note that AMX exhibits zwitterionic behavior, with pKa_1_ = 2.25, pKa_2_ = 7.22, and pKa_3_ = 9.48 [7]. Near neutral pH, the adsorption of AMX is favored by electrostatic interactions (Figure 5b) and diffusion processes associated with the material’s water absorption capacity [44]. In the case of SMX, the presence of an aromatic amine and sulfonamide groups makes its behavior pH dependent. At specific pH values, SMX exists in a neutral state, and its removal relies on both electrostatic interactions and hydrogen bonding [45,46]. Thus, the highest removal of SMX was achieved at pH 4.0, where the molecule is in its neutral form, suggesting that hydrogen bonding (Figure 5b) and diffusion processes played key roles in the adsorption.

### 2.4. Evaluation of Ionic Strength in Antibiotic Removal

**Figure 6:**
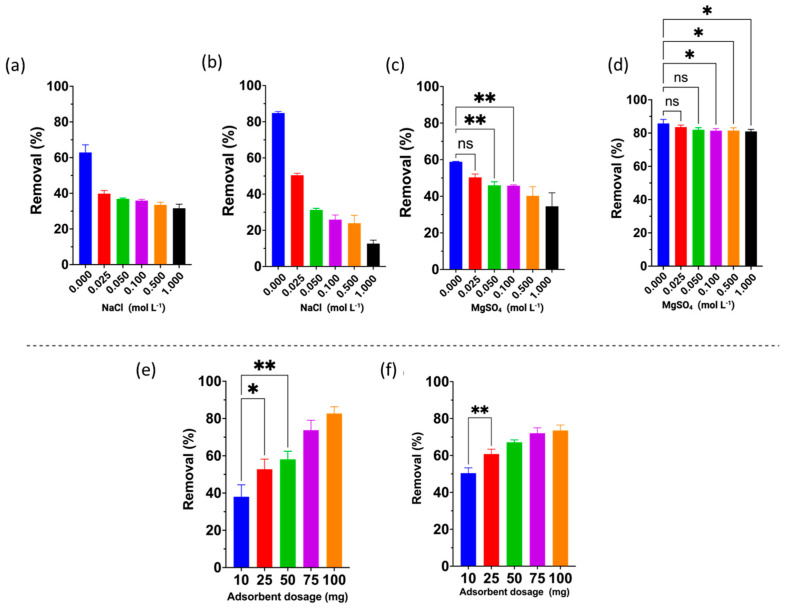
Effect of the ionic strength of monovalent and divalent ions on the removal of the antibiotics (**a**,**c**) sulfamethoxazole and (**b**,**d**) amoxicillin; effect of the adsorbent dose on the removal of (**e**) amoxicillin and (**f**) sulfamethoxazole. Those values that are very significant are not expressed graphically, only those of low significance and those that are not significant are detailed. ** Significant at *p* < 0.05, * Significant at *p* < 0.01, ns = no significant difference.

In general, the presence of dissolved ions in aqueous solutions is reported to act as interfering agents, potentially reducing removal capacity due to various interactions among different species. Thus, the effect of monovalent and divalent ions on the removal capacity of antibiotics was evaluated [47]. For AMX, a significant difference in removal efficiency was observed. As the NaCl concentration increased, the rate of retention decreased (Figure 6b). Given the dual nature of AMX as a charged molecule at pH 6.0, its behavior in solution is impacted by the presence of Na^+^ and Cl^−^ ions. This results in a competitive interaction between the AMX molecule and the active sites of the ECCs, effectively preventing AMX from binding to these sites and, consequently, from exerting any influence on the removal of ions in solution. The rates exceed 80% in the absence of NaCl, falling to 45% when a concentration of 0.025 M NaCl is applied. Further increases in concentration result in a continued decrease in removal, falling to approximately 17% at a concentration of 1.00 M.

In contrast, at pH 4.0, SMX is relatively neutral (Figure 6a), although positively charged species may also be present. Despite being mostly neutral, the interaction between SMX and the CHPs is hindered by chloride ions, which interact with the few positively charged sites on the antibiotic and the protonated amino groups of the CHPs. For both antibiotics, the presence of Na^+^ and Cl^−^ ions play a significant role in the removal process, directly affecting electrostatic interactions and hydrogen bonding between the CHPs and the antibiotics AMX and SMX, ultimately reducing removal capacity.

As in the previous case, an increase in salt concentration leads to a decrease in removal capacity. However, the effect of divalent ions is much less pronounced for AMX (Figure 6d), with removal remaining similar despite the increase in MgSO_4_ concentration. A similar trend is observed for SMX at 0.0025 mol L^−1^ and in the absence of salt (Figure 6c). This effect can be attributed to the interaction of Mg^2^^+^ ions with the -OH groups present in chitosan. Additionally, Mg^2^^+^ ions are smaller than antibiotic molecules, allowing them to diffuse into the structure more easily. As with monovalent ions, increasing the salt concentration reduces removal capacity due to the higher availability of competing ions.

For both antibiotics, the effect of adsorbent dose followed the same trend: removal improved with increasing adsorbent dosage (Figure 6e,f). This behavior is attributed to the higher number of active sites available in the crosslinked CHPs, which enhances the attraction of more antibiotic molecules to the greater quantity of CHPs present in the system. Additionally, it was observed that while higher doses increase removal efficiency, the effect becomes less pronounced at higher adsorbent concentrations due to the saturation of the active adsorption sites [48,49].

### 2.5. Evaluation of Retention Kinetics at Different Temperatures

**Figure 7:**
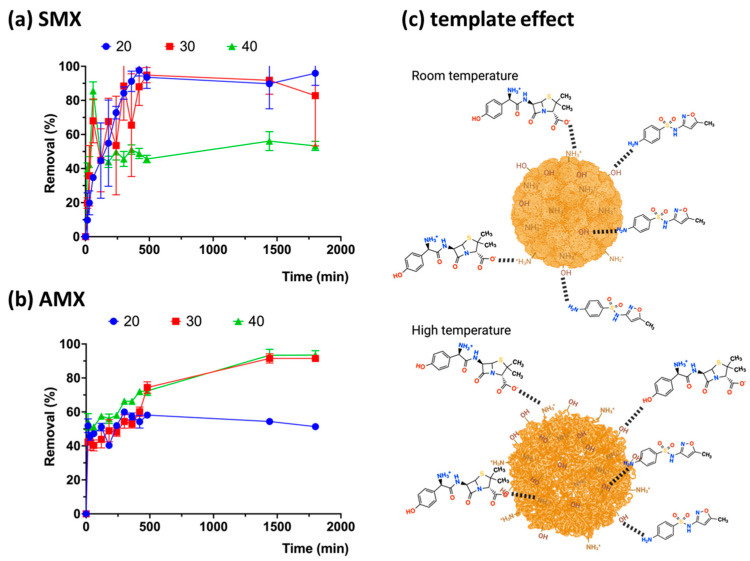
Absorption kinetics of the antibiotics amoxicillin (**a**) and sulfamethoxazole (**b**) by Chi-Glu CHPs as a function of temperature, (**c**) template effect.

Adsorption generally improves with increasing temperature; however, AMX exhibits a reverse effect at 40 °C (Figure 7a) compared to SMX (Figure 7b). SMX shows higher adsorption at elevated temperatures, as the chains of the crosslinked CHPs become more relaxed. Additionally, the smaller molecular size of SMX allows it to penetrate more easily into the structure of the crosslinked CHPs, facilitating stronger interactions [50].

### 2.6. Effect of Initial Antibiotic Concentration at Different Temperatures

**Figure 8:**
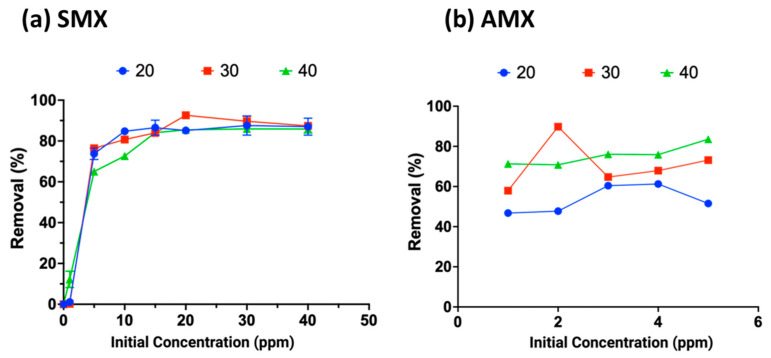
Effect of concentration variation on the adsorption of antibiotics amoxicillin (**a**) and sulfamethoxazole (**b**) by Chi-Glu at different temperatures.

The antibiotics were allowed to interact with the crosslinked CHPs (Chi-Glu5%) for the maximum adsorption time determined in the previous section. The effect varied between the two antibiotics: SMX showed higher adsorption at elevated temperatures, whereas AMX reached similar adsorption percentages around 80% across all temperatures. The removal capacities recorded were 11.65, 11.67, and 11.49 mg g^−1^ of CHPs at 20, 30, and 40 °C, respectively. These values remained consistent regardless of temperature, indicating that the effect is primarily influenced by concentration. Similar values have been reported for cellulose-derived beads (10.8 mg g^−1^) [51], chitosan/biochar beads (7.64 mg g^−1^) [52], and silica nanostructures (24.15 mg g^−1^), as well as other results shown in Table S1, which are directly related to removing AMX and SMX [53]. In each case, adsorption capacity increased with rising temperature (Figure 8a,b), albeit not significantly. This increase can be attributed to the enhanced molecular mobility in solution and the greater flexibility of the polymer chains in the crosslinked CHPs, resulting in an increased number of active adsorption sites [54,55].

Thermodynamic parameters, such as the standard Gibbs free energy change (ΔG°), standard enthalpy change (ΔH°), and standard entropy change (ΔS°), provide insights into the adsorption mechanism, distinguishing between physisorption and chemisorption [56]. This evaluation was performed using Freundlich isotherm models, yielding ΔH° values of 28.38 kJ mol^−1^, 12.39 kJ mol^−1^, and ΔS° 97.19 J mol^−1^ K^−1^, and 33.94 J mol^−1^ K^−1^ for AMX and SMX, respectively (Figures S1 and S2). The results are summarized in Table 1. For AMX, the negative ΔG° values at all temperatures indicate that the adsorption process is spontaneous. Additionally, the decrease in ΔG° with increasing temperature suggests that higher temperatures facilitate adsorption. This phenomenon occurs because the enhanced molecular mobility at elevated temperatures promotes interactions between the CHPs and the antibiotic molecules. The positive ΔH° values indicate that the adsorption of AMX onto CHPs is an endothermic process. Furthermore, the adsorption mechanism is identified as physisorption, as chemisorption generally predominates only when ΔH° values exceed 30 kJ mol^−1^ [57].

For AMX, the positive values indicate the irreversibility and stability of the adsorption process [58], as the antibiotic binds strongly to the active sites of the CHPs. For sulfamethoxazole, both ΔH° and ΔS° values are positive, indicating that, similar to AMX, the adsorption process is endothermic, irreversible, and involves strong binding to the active sites of the CHPs, though to a lesser extent than with AMX. Although these values are positive, they decrease with increasing temperature, suggesting that, as with AMX, higher temperatures enhance adsorption. However, unlike AMX, the adsorption process for sulfamethoxazole is not spontaneous.

## 3. Conclusions

Cationic hydrogel particles (CHPs) are promising adsorbent materials for the treatment of water contaminated with emerging pollutants. The CHPs were characterized using spectroscopic and thermal techniques, confirming the presence of key functional groups. Adsorption of the studied antibiotics occurred at pH 6.0 for AMX and pH 4.0 for SMX, with a contact time of 6 h. While the adsorbent dosage, set at 30 mg, allowed a removal efficiency of 90% for AMX and 60% for SMX, primarily through adsorption processes, with physisorption being the dominant mechanism between the CHPs and the antibiotics. The interactions were affected by the increase in ion concentrations (both monovalent and divalent), with higher ion concentrations resulting in decreased antibiotic adsorption, so that the analysis of the removal was favored in the absence of ionic compounds. The most significant impact was observed for amoxicillin, with a reduction of over 60% in removal efficiency under ion interference. Current and future perspectives highlight the urgent need for improved technologies to treat and remediate water bodies contaminated with antibiotics. In this context, CHPs present a viable alternative for various processes involved in wastewater treatment and a preliminary step for the degradation of these antibiotics.

## 4. Materials and Methods

### 4.1. Reagents

Chitosan (Chi) with low molecular weight, 75–85% deacetylated, was obtained from Sigma-Aldrich; glacial acetic acid for analysis (HAc); sodium hydroxide (NaOH) for analysis; glutaraldehyde (50% in water) for synthesis (Glu); absolute ethanol EMSURE (EtOH); amoxicillin (AMX) and sulfamethoxazole (SMX) of analytical-grade standard (Titripur,); 0.1N HCl standard and 0.1N NaOH standard (Titripur). All reagents were purchased from Sigma-Aldrich Chile, Santiago, Chile.

### 4.2. Synthesis and Optimization of Cationic Hydrogel Particles (CHPs)

**Figure 9:**
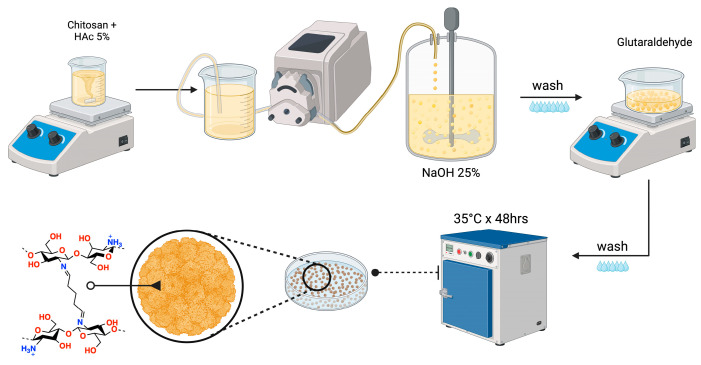
Process for obtaining cationic hydrogel particles.

Chitosan is dissolved in 5% (*w*/*v*) acetic acid, and the viscous solution is stirred until complete dissolution. The solution is then added dropwise into NaOH solutions at 20% and 25%, using a peristaltic pump. CHPs form upon contact between the viscous mixture and the alkaline solution. The mixture is stirred for 30 min, and the CHPs are washed until reaching a neutral pH (Figure 9) [59]. The obtained CHPs are exposed to glutaraldehyde at different concentrations (Table 2) to determine which formulation yields the best results for subsequent analyses.

### 4.3. Characterization

Fourier Transform Infrared Spectroscopy (FTIR): Spectra were obtained to examine the presence of characteristic functional groups of chitosan and its modifications. The samples were recorded in the frequency range of 400 to 4000 cm^−1^ using a Nicolet spectrometer equipped with a DTGS-KBr detector.

Scanning Electron Microscopy (SEM): The surface characteristics of the CHPs were studied using a JEOL-SEM-PROBE CAMECA SU-30 microscope equipped with an EDS detector.

Thermogravimetric Analysis (TGA): Thermogravimetric spectra of the samples were recorded using a NETZSCH 209 F1 Iris thermogravimetric analyzer. Measurements were conducted from room temperature to 550 °C, with a heating rate of 10 °C min^−1^ under a nitrogen atmosphere.

Water Absorption Capacity: A sample of known mass was used to assess water absorption capacity, evaluating the effect over time from 0 to 24 h under agitation.

Point of Zero Charge (PZC) Determination: Fifty milliliters of distilled water was adjusted to pH values between 3 and 11. To each solution, 0.5 g of the adsorbent material was added, and the mixtures were stirred for 48 h at room temperature. The final pH was measured, and the PZC was determined as the point where the final pH curve intersects the diagonal line representing the initial pH.

### 4.4. Removal Studies

**Figure 10:**
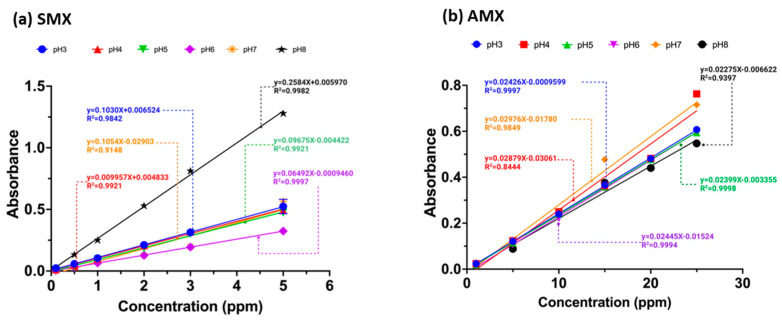
Calibration curves of the antibiotics (**a**) sulfamethoxazole and (**b**) amoxicillin at pH values 3.0–8.0.

Antibiotic solutions were prepared with concentrations of 20 mg L^−1^ for AMX and 5 mg L^−1^ for SMX at pH values of 3, 4, 5, 6, 7, and 8. Each solution was mixed with 30 mg of the adsorbent sample and agitated for 6 h. Afterward, the solutions were measured using a Thermo Fisher Evolution One Plus UV–vis spectrophotometer. Calibration curves (Figure 10a,b) were obtained for each antibiotic at different pH values to determine the removal percentages.

With the pH value determined in the previous section, antibiotic solutions were prepared at different ionic strengths (0.025, 0.050, 0.1, 0.5, and 1 M) using Na^+^, Mg^2^^+^ cations, and Cl^−^, SO_4_^2−^ anions. The solutions were in contact with the CHPs for 6 h in a horizontal shaker and subsequently analyzed using a UV–vis spectrophotometer. For the effect of CHP dosage, 0.005, 0.025, 0.05, 0.075, and 0.1 g of material were used under the optimal experimental conditions described previously.

The effect of temperature and time on the removal process was evaluated at temperatures of 25, 30, 40, and 50 °C and at time intervals of 0, 15, 30, 60, 120, 180, 240, 300, 360, 420, 480, 1440, and 1800 min, following the previously established experimental conditions. To assess the effect of antibiotic concentration at different temperatures, optimal experimental conditions (pH, ionic strength, adsorbent dosage, and contact time) were employed. Thermodynamic parameters (ΔH°, ΔG°, and ΔS°) were also evaluated based on these conditions.

## Figures and Tables

**Table 1:** Gibbs energy values for antibiotics AMX and SMX at different temperatures.

		ΔG (KJ mol^−1^)	
Temperature (K)	293.15	303.15	313.15
AMX	−0.113	−1.085	−2.057
SMX	2.447	2.107	1.768

**Table 2:** Variables for obtaining CHPs.

NaOH (%)	Glutaraldehyde (%)
25	1
	3
	5
	10
	20

## Data Availability

Data are contained within the article.

## Supplementary Materials

The following supporting information can be downloaded at: https://www.mdpi.com/article/10.3390/gels10120760/s1, Figure S1. Determination of thermodynamic parameters using the Freundlich model for the antibiotic amoxicillin, Figure S2. Determination of thermodynamic parameters using the Freundlich model for the antibiotic Sulfamethoxazole, Table S1. Comparison of the adsorption capacity of the various adsorbents from the literature and with this study [60,61,62,63,64,65,66,67,68,69,70].
