# Supplementary material for: Development of Variable Charge Cationic Hydrogel Particles with Potential Application in the Removal of Amoxicillin and Sulfamethoxazole from Water
Source: Gels. 2024 Nov 23;10(12):760. doi: 10.3390/gels10120760 (PMC11675227; doi:10.3390/gels10120760)
Supplement: Supplementary file 1 [file gels-10-00760-s001.zip › gels-3282699-supplementary.pdf]

Supporting information for:

Development of Variable Charge Cationic Hydrogel Particles with Potential Application in the Removal of Amoxicillin and Sulfamethoxazole From Water

Francisca L. Aranda [1-2], Manuel F. Meléndrez [3] , Mónica A. Pérez [2], Bernabé L. Rivas [2], Eduardo D. Pereira [4] and Daniel A. Palacio [2*]

[1] Departamento de Ingeniería de Materiales, Facultad de Ingeniería, Universidad de Concepción. Edmundo Larenas 315. Box 160 – C, Concepción, Chile
[2] Departamento de Polímeros, Facultad de Ciencias Químicas, Universidad de Concepción, Casilla 160-C, Concepción, Chile
[3] Facultad de Ciencias para el cuidado de la Salud, Universidad San Sebastián, Campus Las Tres Pascualas, Lientur 1457, Concepción 4060000, Chile
[4] Departamento de Química Analítica e Inorgánica, Facultad de ciencias Químicas, , Universidad de Concepción, Casilla 160-C, Concepción, Chile

*Correspondence: dapalacio@udec.cl

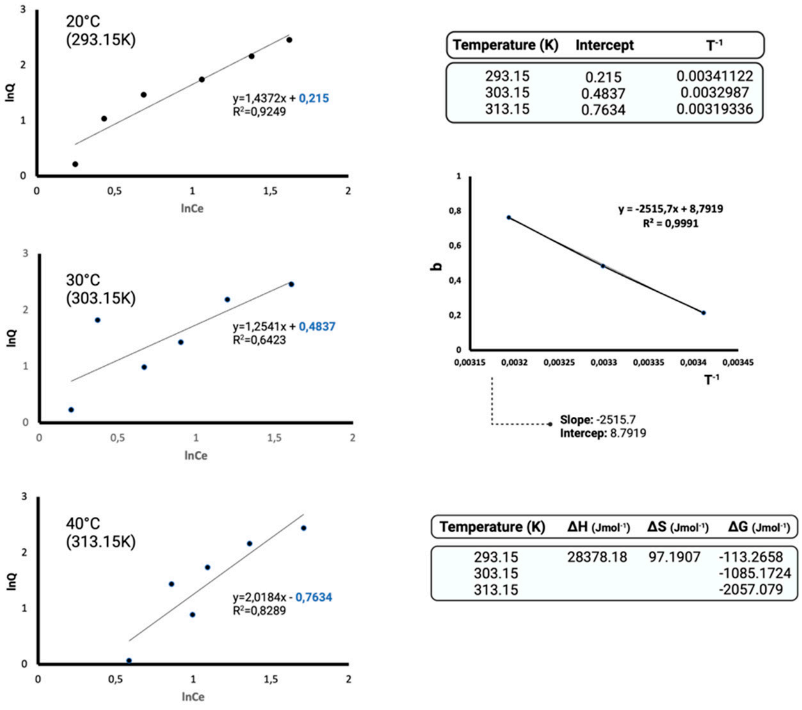

Figure S1. Determination of thermodynamic parameters using the Freundlich model for the antibiotic amoxicillin

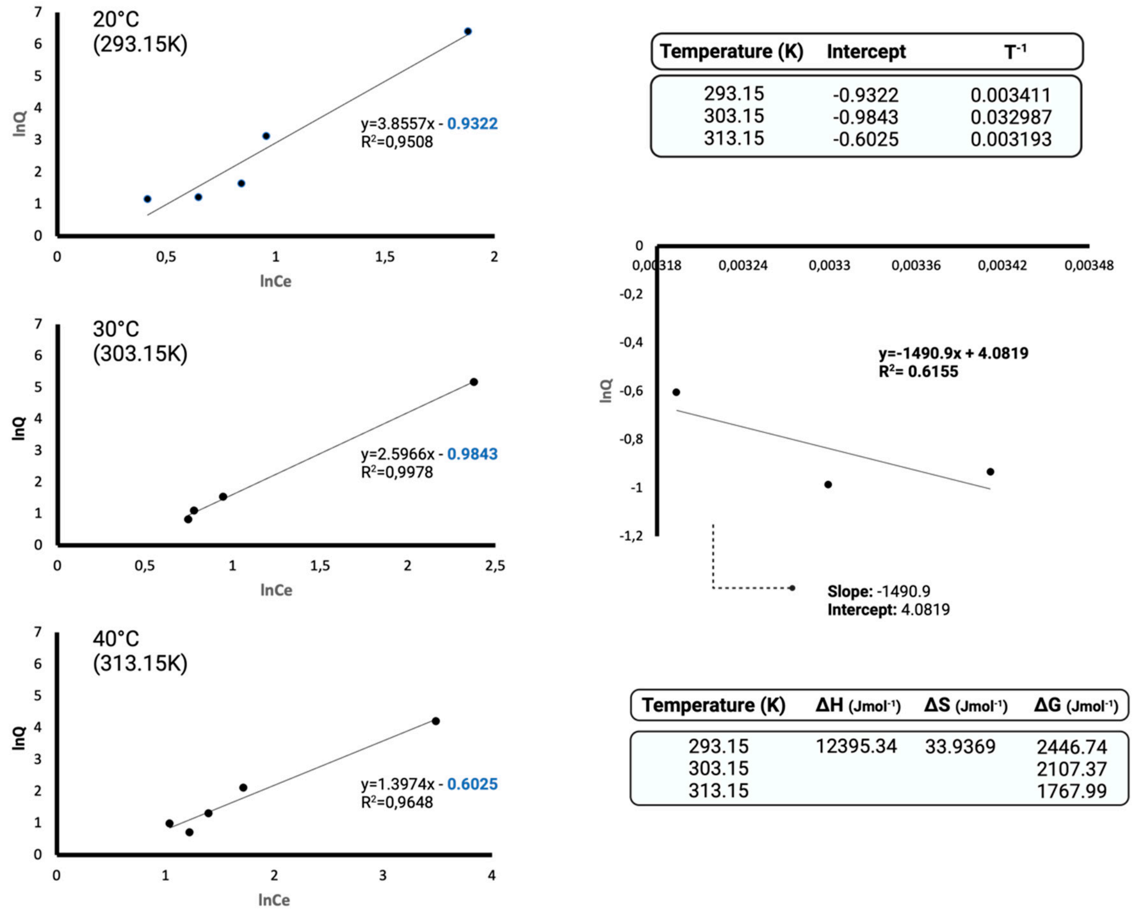

Figure S2. Determination of thermodynamic parameters using the Freundlich model for the antibiotic Sulfamethoxazole

Table S1. Comparison of the adsorption capacity of the various adsorbents from the literature and with this study.

Antibiotics	Adsorbent	Maximum adsorption capacity mg g^{-1}
Amoxicillin	Chi-Bentonite [1]	51.9
	Chitosan-beads [2]	8.7
	Bentonite [3]	43.37
	Iron-oxide/activated carbon/chitosan nanocomposite [4]	526.31
	Alkylated chitosan [5]	185.6
	Chitosan/microalgae biocomposites [6]	125.6
	Alginate composites[7]	71.3

	CHPs (**This study**)	11.64
Sulfamethoxazole	Chi-PVA/C_3N_4/Glu[8]	5.8
	Chitosan-CNT [9]	25.17
	Biochar from corncob [10]	425
	Ag_2O nanoparticles[11]	277.85
	Microcrystalline cellulose – Chitosan[12]	45
	OB	3.49
	SCB	7.65
	Chitosan-OB	7.24
	Chitosan-SCB [13]	14.73
	CHPs (T**his study**)	1.4

1. Yeo, J.Y.J., et al., *Experimental and modelling study of adsorption isotherms of amoxicillin, ampicillin and doripenem on bentonite-chitosan composite*. South African Journal of Chemical Engineering, 2023. **43**(1): p. 38-45.
2. Adriano, W.S., et al., *Adsorption of amoxicillin on chitosan beads: Kinetics, equilibrium and validation of finite bath models*. Biochemical Engineering Journal, 2005. **27**(2): p. 132-137.
3. Putra, E.K., et al., *Performance of activated carbon and bentonite for adsorption of amoxicillin from wastewater: Mechanisms, isotherms and kinetics*. Water Research, 2009. **43**(9): p. 2419-2430.
4. Danalıoğlu, S.T., et al., *Efficient removal of antibiotics by a novel magnetic adsorbent: Magnetic activated carbon/chitosan (MACC) nanocomposite*. Journal of Molecular Liquids, 2017. **240**: p. 589-596.
5. Palacio, D.A., et al., *Antibiotics removal using a chitosan-based polyelectrolyte in conjunction with ultrafiltration membranes*. Chemosphere, 2020. **258**.
6. Mirizadeh, S., et al., *Efficient removal of tetracycline, ciprofloxacin, and amoxicillin by novel magnetic chitosan/microalgae biocomposites*. Separation and Purification Technology, 2024. **329**: p. 125115.
7. Pinheiro, C.P., et al., *Adsorption of amoxicillin by chitosan and alginate biopolymers composite beads*. Environmental Science and Pollution Research, 2024.
8. Zhou, A., et al., *Functionally-Designed Chitosan-based hydrogel beads for adsorption of sulfamethoxazole with light regeneration*. Separation and Purification Technology, 2022. **293**: p. 120973.
9. Khumalo, S.M., B.F. Bakare, and S. Rathilal, *Single and multicomponent adsorption of amoxicillin, ciprofloxacin, and sulfamethoxazole on chitosan-carbon nanotubes hydrogel beads from aqueous solutions: Kinetics, isotherms, and thermodynamic parameters*. Journal of Hazardous Materials Advances, 2024. **13**: p. 100404.
10. Li, Y., et al., *Influence of adsorption sites of biochar on its adsorption performance for sulfamethoxazole*. Chemosphere, 2023. **326**: p. 138408.

11. El Messaoudi, N., et al., *Green synthesis of Ag2O nanoparticles using Punica granatum leaf extract for sulfamethoxazole antibiotic adsorption: characterization, experimental study, modeling, and DFT calculation*. Environmental Science and Pollution Research, 2023. **30**(34): p. 81352-81369.
12. Mashile, P.P., *Biopolymer-based nanocomposite as recyclable adsorbents for removal of pollutants in wastewater treatment*. 2023, University of Johannesburg.
13. Son Tran, V., et al., *New chitosan-biochar composite derived from agricultural waste for removing sulfamethoxazole antibiotics in water.* Bioresource Technology, 2023. **385**: p. 129384.
